# Angiomatosis of the Breast: Unveiling a Rare and Unusual Vascular Puzzle

**DOI:** 10.7759/cureus.96346

**Published:** 2025-11-07

**Authors:** A Aishwarya Teja, Anagha Damodaran, Ashwini Pitambra, Jitendra S Nigam, Sunil Kumar

**Affiliations:** 1 Pathology and Laboratory Medicine, All India Institute of Medical Sciences, Bibinagar, Bibinagar, IND; 2 General Surgery, All India Institute of Medical Sciences, Bibinagar, Bibinagar, IND

**Keywords:** benign neoplasm, histopathology, immunohistochemistry, mammary angiomatosis, vascular neoplasms

## Abstract

Angiomatosis of the breast is a very rare benign vascular lesion that often resembles malignant vascular tumors both clinically and radiologically. Accurate recognition is crucial to avoid misdiagnosis and unnecessary interventions. We report the case of a 35-year-old female who presented with a 20-year history of recurrent, painless enlargement of the left breast. A nipple-sparing mastectomy was performed, and the specimen, measuring 29.5 × 17.5 × 2.5 cm, revealed a diffuse spongy lesion with multiple cystic spaces. Microscopically, the lesion was composed of anastomosing vascular channels of variable caliber surrounding but not invading breast lobules. Immunohistochemistry demonstrated strong nuclear positivity for FLI1 and cytoplasmic positivity for CD31 and CD34, with weak or absent CD34 expression in some vascular spaces. The Ki-67 proliferation index was <2%. These findings suggest the diagnosis of breast angiomatosis. The postoperative recovery was smooth, and no recurrence was observed after four months of follow-up.

These cases must be distinguished from low-grade angiosarcoma, which shares overlapping histological features. Features such as the absence of cytologic atypia, a low proliferative index, and supportive immunohistochemical findings help confirm the diagnosis. Complete surgical excision with clear margins is essential, as incomplete excision carries a risk of local recurrence. This report highlights the importance of thorough histopathological and immunohistochemical evaluation in differentiating angiomatosis from malignant vascular tumors of the breast.

## Introduction

Angiomatosis of the breast is an exceptionally rare benign vascular lesion, with only a handful of cases reported so far. These lesions typically manifest as slow, painless breast enlargements and tend to recur, making mastectomy the definitive treatment option [1]. While imaging can suggest the presence of a vascular lesion, it is often challenging to distinguish between benign and malignant forms [2,3]. Therefore, histopathological examination is essential for accurate diagnosis and classification.

## Case presentation

Clinical impression and management

A 35-year-old female presented to the outpatient department of surgery with a complaint of recurring enlargement of the left breast for 20 years. The enlargement of the breast was not associated with pain, trauma, nipple discharge, palpable lump, or skin changes. She had undergone reduction mammoplasty approximately 10 years earlier, but the breast enlargement had persisted and progressed to its current size. A nipple-sparing mastectomy was done, and the specimen was sent for histopathological analysis.

Histopathological findings

**Figure 1:**
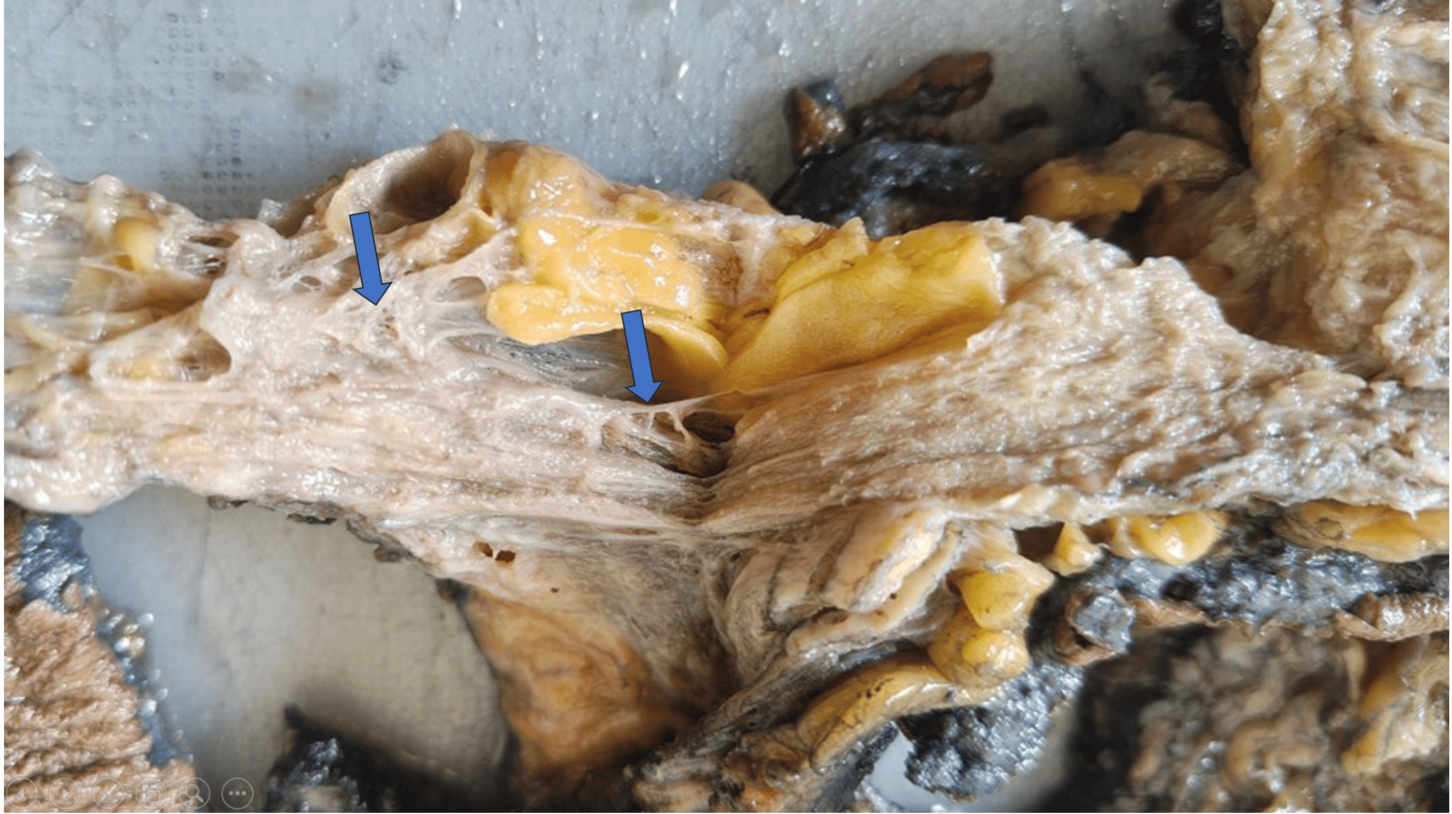
Gross specimen Angiomatosis with an ill-defined diffuse lesion with a cystic and spongy cut surface

A specimen of left mastectomy was received, measuring 29.5 x 17.5 x 2.5 cm. The skin overlying the specimen was unremarkable. There was no grossly palpable mass. The cut surface showed a 16.5 x 15 x 2 cm diffuse lesion with spongy areas and multiple cystic spaces ranging from 0.3 to 1 cm in diameter (Figure 1).

**Figure 2:**
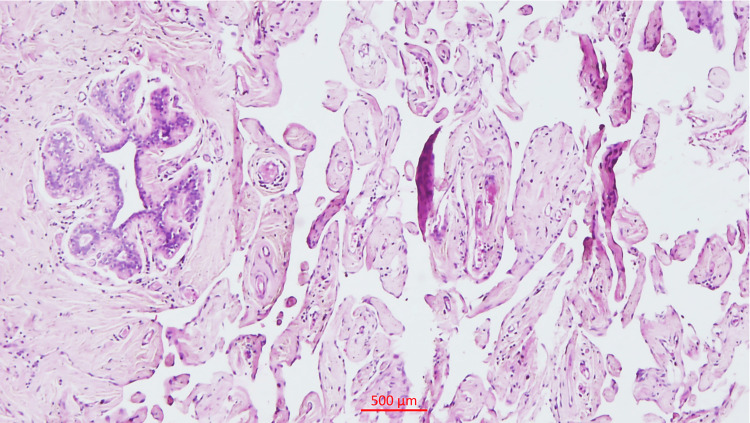
Histology Anastomosing vascular channels lined by flat inconspicuous endothelial cells, sparing intralobular stroma (5x)

The intervening septa of the cystic spaces were thin and delicate, with few of the spaces containing blood. On microscopy, diffuse anastomosing vascular channels of varying calibre were observed, with some containing red blood cells and others being empty, surrounding the lobules of the breast while sparing the intralobular stroma. The vascular channels are lined by flat endothelial cells without atypia (Figure 2).

The surrounding stroma appeared focally fibrosed and showed few lymphoid aggregates.

Immunohistochemical findings

**Figure 3:**
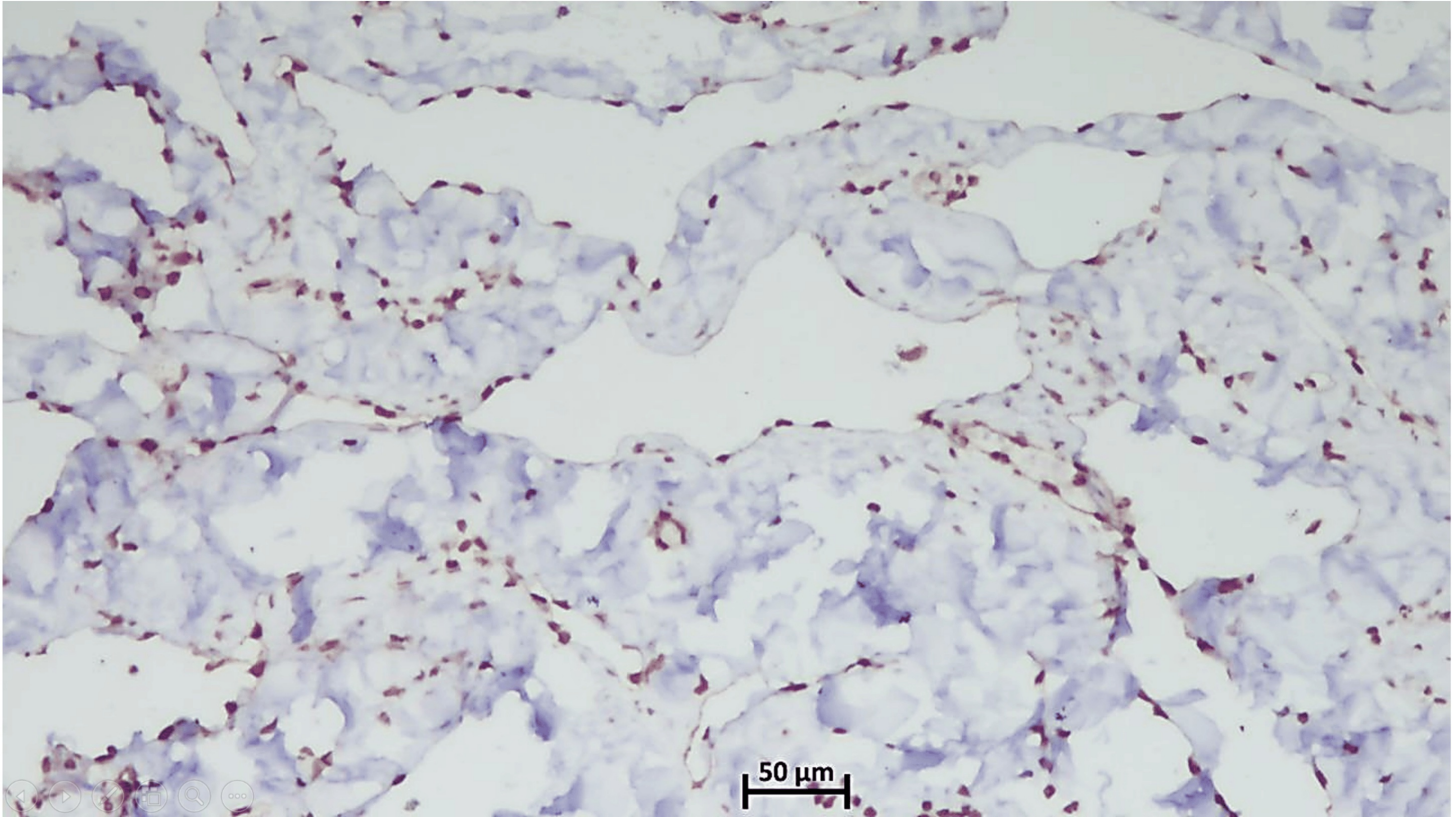
FLI 1 Highlighting both hemangiomatous and lymphangiomatous components (10x) FLI: friend leukemia integration

**Figure 4:**
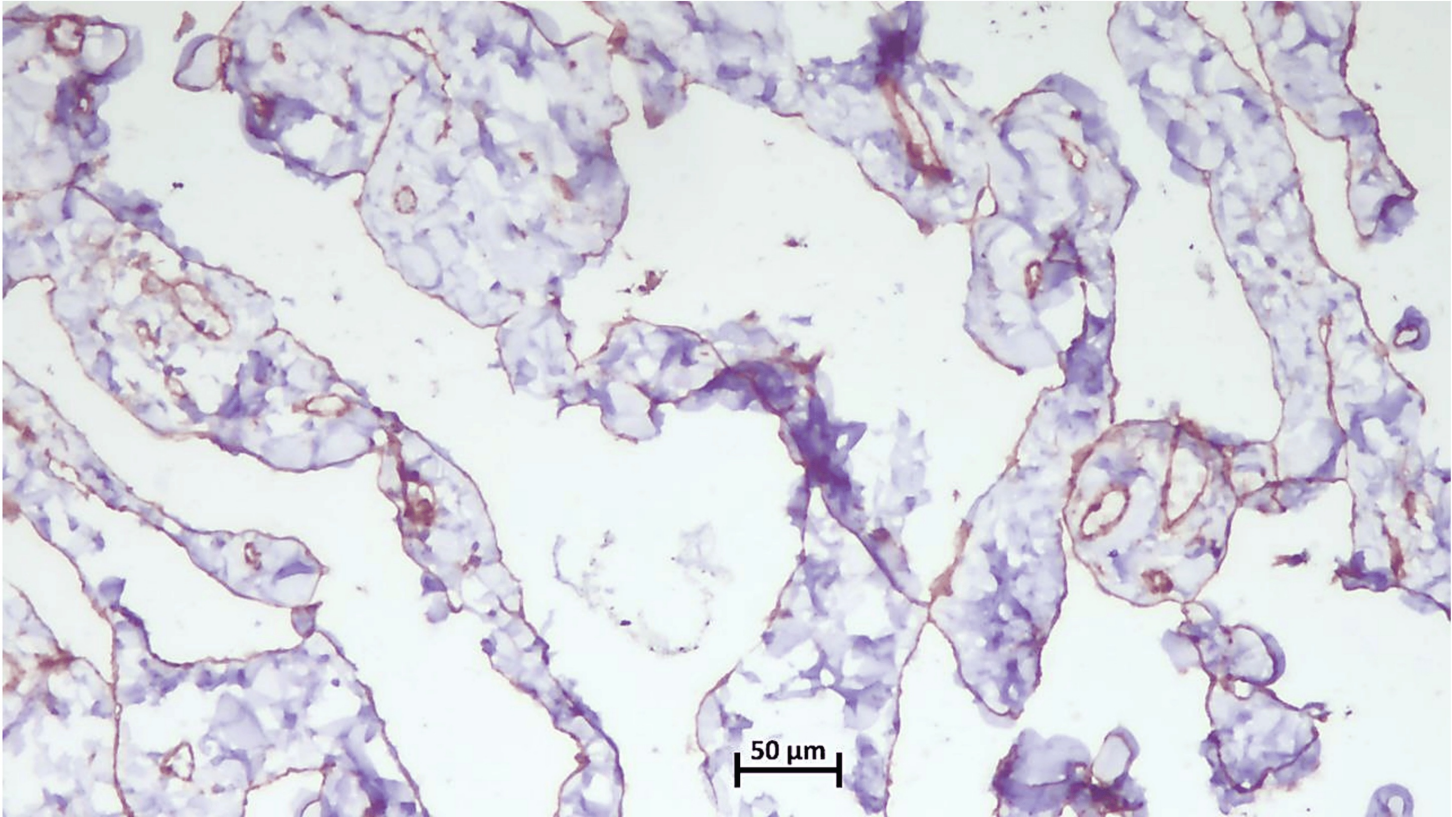
CD31 Highlighting both hemangiomatous and lymphangiomatous components (10x) CD: cluster of differentiation

**Figure 5:**
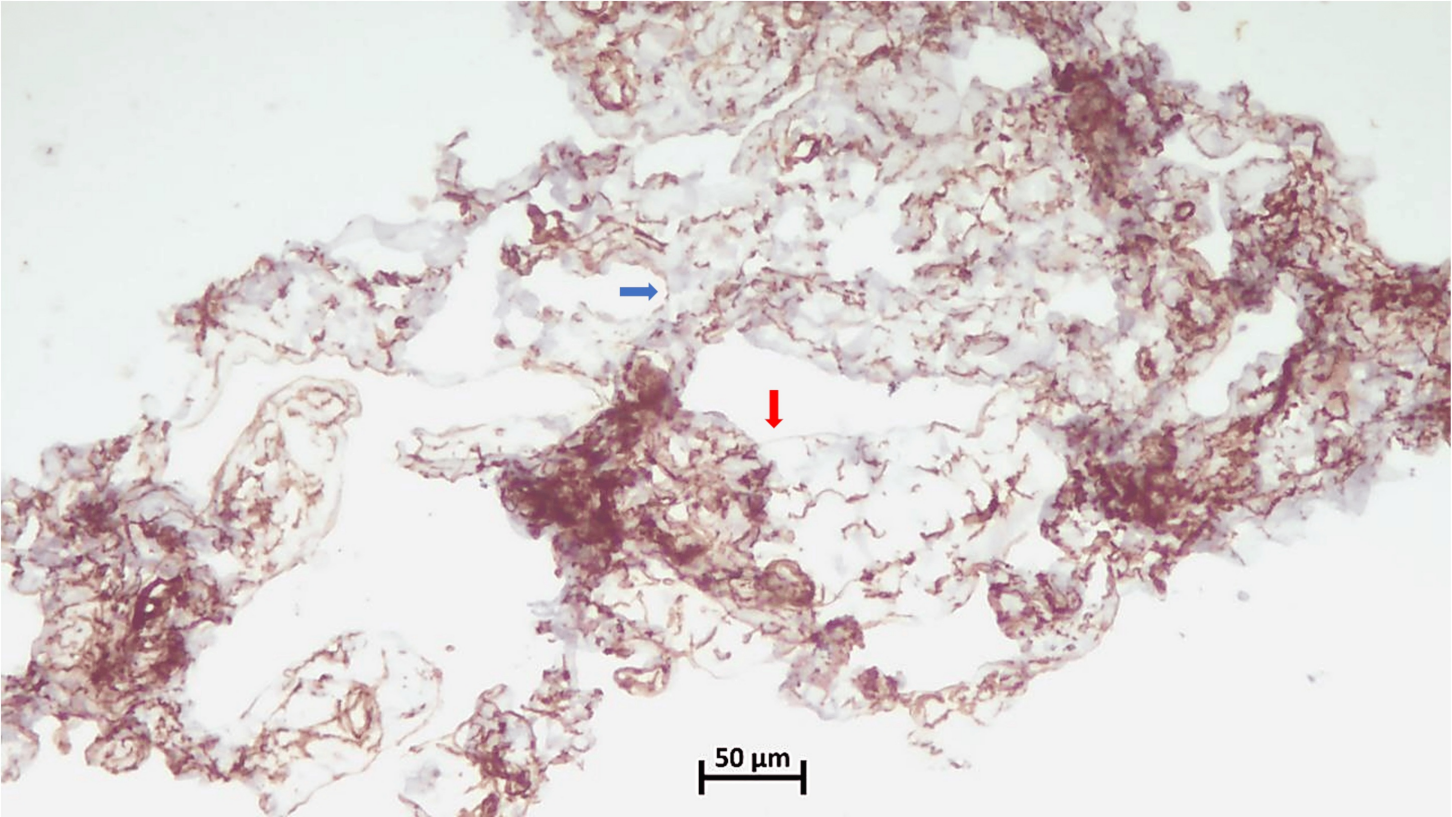
CD34 Weak patchy positivity in the hemangiomatous (red arrow), while negative in the lymphangiomatous component (blue arrow) (10x) CD: cluster of differentiation

**Figure 6:**
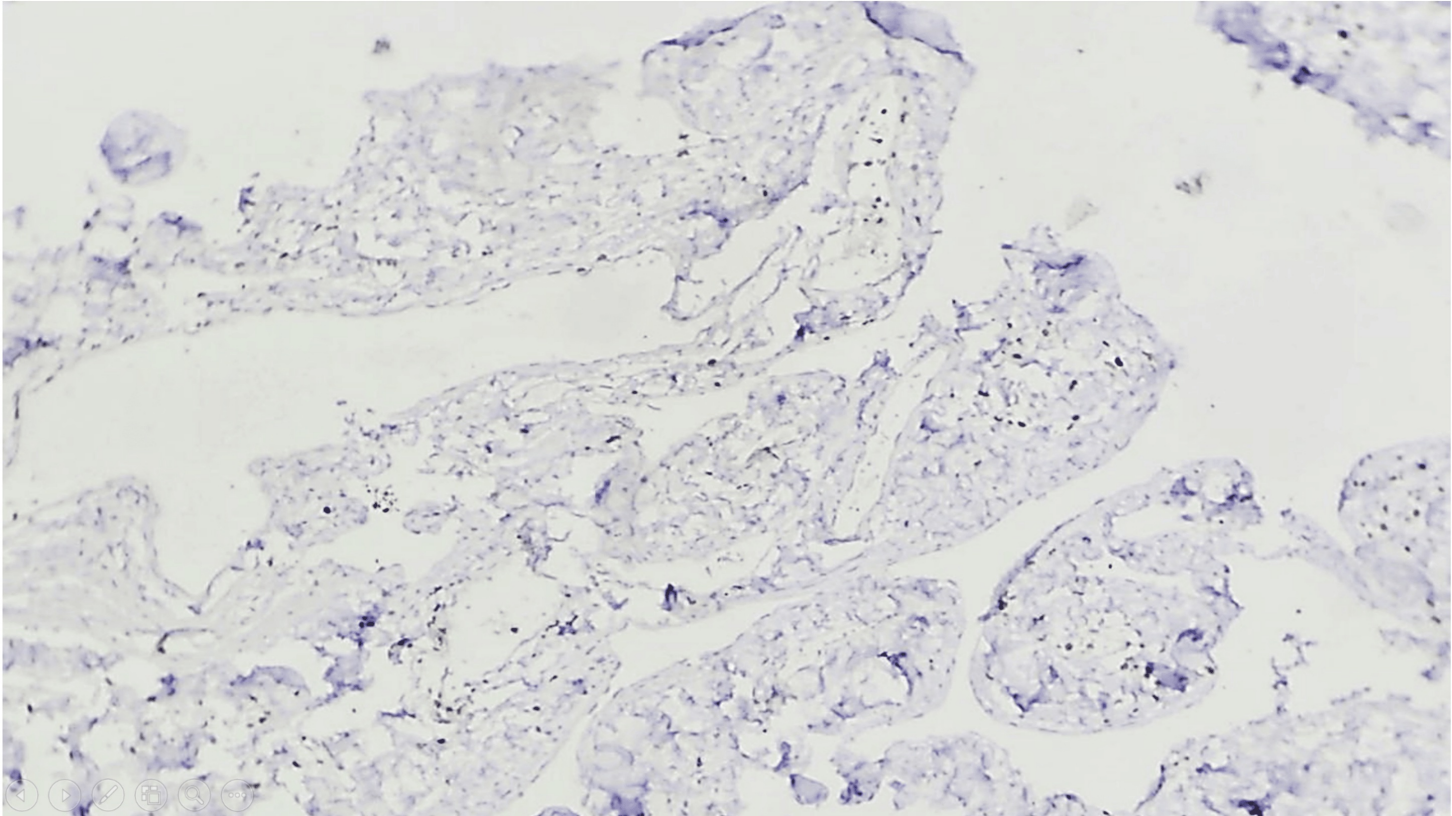
Ki67 Proliferation index is low (<2%) (10x)

On immunohistochemistry (IHC), some of the vascular channels showed strong and diffuse nuclear positivity for FLI1 (friend leukemia integration 1) (Figure 3) and cytoplasmic positivity for CD31 (cluster of differentiation 31) (Figure 4) and CD34 (cluster of differentiation 34) (Figure 5) in the lining endothelial cells. Few vascular spaces showed no immunoreactivity or weak positivity for CD34. The Ki-67 index was low (Figure 6).

Outcome

The patient was followed up weekly for a period of four months, and it was uneventful.

## Discussion

Vascular tumors of the breast are uncommon, and of those, the majority are usually malignant [1]. Benign vascular lesions are extremely rare and can be congenital or acquired [4]. Angiomatosis predominantly affects young females, typically between 18 and 59 years of age, with an average age of onset around 37 years (Table 1).

**Table 1:** Comprehensive review of published cases of breast angiomatosis MRI: magnetic resonance imaging; BI-RADS: Breast Imaging Reporting and Data System

Author	Year	Country	Article type	Age in years	Gender	Clinical details	Side	Quadrant	Radiology: ultrasonography/mammography/MRI	Tumour size in cm
Sundaram [1]	2012	India	Case report	18	Female	Rapidly enlarging mass	Left	Not available	Not available	14 x 12 x 8
Krishna et al. [2]	2015	India	Case report	18	Female	Painless mass	Right	Not available	Not available	9 x 6 x 5
Mekhail et al. [3]	2017	USA	Case report	28	Female	Asymptomatic (incidental)	Right	9 o'clock	MRI: mass with heterogeneous contrast uptake with irregular margins	2.3
Ginter et al. [4]	2019	USA	Case series	58	Female	Breast mass	Left	Axilla	Not available	9
	2019	USA	Case series	Not available	Female	Breast mass	Left	Upper outer quadrant	Ultrasonography: fluid collection with an echoic component and a heterogeneous, loculated/solid component	3.5
	2019	USA	Case series	19	Female	Palpable irregularity	Right	Upper outer quadrant	Ultrasonography: slightly macrolobulated, ovoid mixed echogenic mass	3.4
	2019	USA	Case series	51	Female	Calcification	Left	Not available	Mammography: calcification	Not available
	2019	USA	Case series	56	Female	Breast mass	Right	Upper inner quadrant	Ultrasonography: conglomerate cystic mass	2.8
	2019	USA	Case series	41	Female	MRI enhancement	Left	Not available	MRI: breast enhancement	Not available
	2019	USA	Case series	37	Female	Breast mass	Left	Not available	Not available	Not available
	2019	USA	Case series	56	Female	MRI enhancement	Right	Upper outer quadrant	Ultrasonography: hypoechoic irregular mass; MRI: Irregular enhancing mass	2
Morrow et al. [5]	1988	USA	Case report	19	Female	Massive enlargement of the breast	Right	All quadrants	MRI: increased signal intensity on T2-weighted imaging	15
Rosen [6]	1985	USA	Case series	59	Female	Breast mass	Right	Not available	Ultrasonography: spongy, focally hemorrhagic mass with no defined margins	9 x 5
	1985	USA	Case series	39	Female	Congenital cavernous vascular tumor with recurrent angiomatosis of the breast	Right	Upper inner and lower inner quadrants	Ultrasonography: breast mass	Not available
	1985	USA	Case series	19	Female	Breast mass	left	Upper outer quadrant	Ultrasonography: breast mass	9.3 x 5.7 x 4
Ciurea et al. [7]	2014	Romania	Case report	34	Female	Breast enlargement, mild tenderness, and mild nipple retraction	Left	Not available	Not available	Not available
Anselmi et al. [8]	2025	Brazil	Case report	22	Female	Ulcerated mass	Right	Junctions of lower quadrants	Ultrasonography: fibroglandular tissue, BI-RADS 1 with no cystic or solid nodules within the lesion	5 x 4
Shirley et al. [9]	2002	Africa	Case report	7	Male	Breast mass	Unilateral	Not available	Not available	Not available
Present case	2025	India	Case report	35	Female	Massive enlargement of the breast	Left	All quadrants	Not available	16.5 x 15 x 2

The most common presentation is a palpable breast mass (37.5%), with variations including painless enlargement, rapidly growing mass, incidental imaging detection, and massive breast enlargement (Table 1). In the present case, the patient was 35 years old and exhibited recurrent breast enlargement without a palpable mass. There is no predilection for either side; however, the upper outer quadrant is most frequently involved.

Radiologically, ultrasonography is the most used modality, on which diverse features were reported as solid hypoechoic masses, spongy hemorrhagic patterns, and heterogeneous vascular lesions (Table 1). MRI has been reported in a few cases, showing heterogeneous enhancement with irregular margins and increased signal intensity on T2-weighted imaging. Some authors regard MRI as a sufficient standalone tool for suggesting the diagnosis of angiomatosis [3-5]. The calcification on mammography was also reported in one case [4]. In the reported cases of angiomatosis, the size of the lesions varied from 2 cm to 15 cm, with a mean size of 7.4 cm (Table 1). Most lesions were ill-defined and diffuse, although some cases were well-defined, exhibiting a cystic and spongy cut surface, and may have been partially filled with serosanguinous fluid [5]. In the present reported case, the lesion measured 16.5 x 15 x 2 cm and appeared spongy and cystic on the cut surface, with a few spaces containing blood.

Microscopically, angiomatosis is distinguished by relatively evenly distributed, anastomosing vascular spaces of varied diameter, which are bordered by flat, inconspicuous endothelial cells that lack atypia, tufting, or mitosis [1-9 ]. The blood vessels spread out widely through the breast tissue and fat, surrounding but not entering the areas between the lobules [1-9]. The lesion may also contain lymphatic channels [1-2,5-6]. In our case, immunohistochemical analysis revealed strong positivity for CD31 and FLI1, highlighting the endothelial cells of both hemangiomatous and lymphangiomatous components. In contrast, CD34 exhibited weak patchy positivity in the hemangiomatous component but was negative in the lymphangiomatous component, while the Ki67 index was low at less than 2%.

Only a few studies have demonstrated CD31 positivity, D2-40 positivity, and low Ki-67 indices (<2%) in breast angiomatosis, findings that were also observed in our case [2,8], distinguishing it from low-grade angiosarcoma. It is critical to distinguish this angiomatosis from a low-grade angiosarcoma of the breast. The histologic appearance of angiomatosis can be identical to that of low-grade angiosarcoma; both encompass anastomosing channels and an infiltrative pattern [1,8]. Angiosarcoma shows a pattern of small blood vessel channels that branch out in lobes, while angiomatosis has a more spread pattern. The endothelial nuclei of angiomatosis seem bland compared to the varying degree of cytologic atypia found in the endothelial nuclei of angiosarcoma [1,3,4,8].

This uncommon lesion might recur after surgery; however, no instances of metastasis or malignant transformation have been reported [2]. As a result, total surgical excision with clear margins is necessary, and mastectomy may be required in certain individuals due to the lesion's diffuse nature [3-5].

## Conclusions

Breast angiomatosis is an uncommon benign vascular lesion that poses notable diagnostic and therapeutic challenges. This report contributes to the sparse literature on breast angiomatosis and underscores the necessity of including this uncommon entity in the differential diagnosis of breast vascular lesions. The favorable prognosis after complete excision, along with the lack of malignant potential, highlights the importance of accurate diagnosis for effective patient management. Regular surveillance is encouraged because of the potential for local recurrence, and further reporting of similar cases will enhance the overall understanding of this condition. At our four-month follow-up, there was no evidence of recurrence, indicating successful complete excision.
